# High expression of HLA-E in colorectal carcinoma is associated with a favorable prognosis

**DOI:** 10.1186/1479-5876-9-184

**Published:** 2011-10-27

**Authors:** Maria Benevolo, Marcella Mottolese, Elisa Tremante, Francesca Rollo, Maria Grazia Diodoro, Cristiana Ercolani, Isabella Sperduti, Elisa Lo Monaco, Maurizio Cosimelli, Patrizio Giacomini

**Affiliations:** 1 Department of Pathology, Regina Elena National Cancer Institute, Via E. Chianesi 53, 00144 Rome, Italy; 2 Laboratory of Immunology, Regina Elena National Cancer Institute, Via delle Messi d'Oro 156, 00158 Rome, Italy; 3 Biostatistics Unit, Istituti Fisioterapici Ospitalieri, Via E. Chianesi 53, 00144 Rome, Italy; 4 Hepato-Biliary-Pancreatic Surgery, Regina Elena National Cancer Institute, Via E. Chianesi 53, 00144 Rome, Italy

## Abstract

**Background:**

Human Leukocyte Antigen (HLA)-E is a non-classical class I HLA molecule that can be stabilized by ligands donated by other classical (HLA-A, -B, -C) and non-classical (HLA-G) family members. HLA-E engages a variety of immune receptors expressed by cytotoxic T lymphocytes (CTLs), Natural killer (NK) cells and NK-CTLs. In view of the opposing outcomes (activation or inhibition) of the different HLA-E receptors, the preferred role (if any) of HLA-E expressed *in vivo *on tumor cells remains to be established.

**Methods:**

Taking advantage of MEM-E/02, a recently characterized antibody to denatured HLA-E molecules, HLA-E expression was assessed by immunohistochemistry on an archival collection (formalin-fixed paraffin-embedded) of 149 colorectal primary carcinoma lesions paired with their morphologically normal mucosae. Lymphoid infiltrates were assessed for the expression of the HLA-E-specific, inhibitory, non-rearranging receptor NKG2A.

**Results:**

High HLA-E expression did not significantly correlate with the expression of classical HLA-B and HLA-C molecules, but it did correlate with high expression of its preferential ligand donor HLA-A. In addition, it correlated with lymphoid cell infiltrates expressing the inhibitory NKG2A receptor, and was an independent predictor of good prognosis, particularly in a subset of patients whose tumors express HLA-A levels resembling those of their paired normal counterparts (HLA-A). Thus, combination phenotypes (HLA-E^lo-int^/HLA-AE and HLA-E^hi^/HLA-AE) of classical and non-classical class I HLA molecules mark two graded levels of good prognosis.

**Conclusions:**

These results suggest that HLA-E favors activating immune responses to colorectal carcinoma. They also provide evidence in humans that tumor cells entertain extensive negotiation with the immune system until a compromise between recognition and escape is reached. It is implied that this process occurs stepwise, as predicted by the widely accepted 'immunoediting' model.

## Background

Human Leukocyte Antigen (HLA)-E is a cell surface, non-classical Major Histocompatibility class I molecule recognized by immune receptors expressed by cytotoxic T lymphocytes (CTLs), Natural Killer (NK) cells, and the more recently described subset of NK-CTLs. These receptors are either inhibitory or activating [1-3].

Inhibition, on the one hand, results from the engagement of the NKG2A receptor with HLA-E heavy chains that have been stabilized upon heterotrimeric assembly with their light chain subunit, called β_2_-microglobulin (β_2_m), and peptide ligands derived from the signal sequences of 'permissive' class I heavy chains, both classical (HLA-A, -B, -C) and non-classical (HLA-G). Activation, on the other hand, results from: (a) the competitive relief of NKG2A-mediated inhibition upon HLA-E assembly with peptides from donor proteins other than HLA class I; (b) the direct engagement of the activating NKG2C receptor isoform; and (c) antigen-specific recognition through the T cell receptor (TcR) expressed by NK-CTLs [1-4]. Balancing and integration of opposing signals (often dubbed activation-inhibition) is not unique of HLA-E, but is indeed widely adopted to control cytotoxic responses and regulate complex immune networks. Thus, HLA-E may provide key information to understand how virus-infected [5] and tumor cells walk the thin line between immune surveillance and immune evasion.

HLA-A, -B, -C down-regulation has been viewed as a major subterfuge to deceive T cells [6] for some time now, but it is unlikely to provide a comprehensive explanation of immune evasion, since it impairs ligand donation to HLA-E and the direct engagement of inhibitory NK receptors [1,7-9]. Accordingly, several immunohistochemical studies failed to confirm an association between HLA-A, -B, - C loss and poor prognosis [10-12], and our own studies were consistent with activation-inhibition models [13-16]. We showed that early-passage melanoma, breast carcinoma, and lung carcinoma cells, like virus-infected cells, avoid both extremes of overly low or high HLA class I expression, which would expose them to lysis by NK and CTLs, respectively. Similar 'low profile' HLA phenotypes were also observed *in vivo*, in colorectal carcinoma lesions, and were associated with a favorable prognosis, whereas extreme down-and up-regulation with respect to the normal autologous mucosa were rare and associated with a poor prognosis, particularly when involving the HLA-A locus [16]. Possibly, these altered HLA phenotypes mark tumor cells refractory to immune elimination.

In an effort to characterize monoclonal antibodies (mAbs) to HLA-E, we found that MEM-E/02 binds a linear epitope highly restricted to the HLA-E polypeptide and fully available upon denaturation [17]. Using MEM-E/02, we recently observed that HLA-E is constitutively co-expressed with HLA-A, -B, -C molecules in a fraction of neoplastic tissues and on the surface of most cultured tumor cell lines. In these conditions, HLA-E is functional [18]. In the present report, we describe the use of MEM-E/02 on archival collections of formalin-fixed/paraffin-embedded colorectal adenocarcinoma tissues and their paired, morphologically normal mucosae. We found that overall HLA-E expression is a significant prognosticator, and in combination with HLA-A expression and the presence of NKG2A infiltrating lymphoid cells provides a clue to understand tumor immune surveillance *in vivo*.

## Methods

### Patients and histological specimens

Patients (149 cases, 71 men and 78 women, median age 64, range 34-90 years) were radically resected for primary colorectal adenocarcinoma between 1988 and 2000. Pathological staging [19], performed according to the American Joint Committee on Cancer (AJCC)/International Union Against Cancer (UICC), identified 98 and 51 tumors at stages II and III, respectively. The latter were treated by adjuvant chemotherapy. According to the WHO classification, well (G1), moderately (G2), and poorly (G3) differentiated tumors were 10, 114, and 25, respectively. T2, T3 and T4 tumors were 26, 63, and 60, respectively. The present archival tissue collection largely overlaps (*n *= 126) with that previously described [16]. Uninvolved mucosal tissues were obtained from the same tissue block as the tumor lesion in 108 cases, and from a distinct tissue block in 41 cases. No patients were lost to clinical follow-up (continuously updated), and the median follow-up was 71.0 (range 3.0 - 175.1) months, with 45 relapses and 37 deaths due to cancer-related causes. This study was approved by the Ethical Committee.

### Antibodies and Immunohistochemistry

MEM-E/02 is the only available mAb to HLA-E with an extensive biochemical characterization. It binds a linear epitope present on HLA-E but not HLA-A, -B, -C, -F or -G heavy chains [17]. HCA2 binds all the denatured HLA-A alleles except A24, and crossreacts with the rare HLA-B73 allele, HLA-G, HLA-E and HLA-F [20,21]. However, HLA-E and HLA-F bind weakly and almost exclusively under native conditions [21], and HLA-G accounts for less than 5% of the total HCA2 reactivity in a large cell panel [15]. HC10 binds HLA-B and weakly crossreacts with HLA-C, whereas L31 binds HLA-C and a few crossreacting HLA-B alleles [20,22,23]. CD8 and CD56 were identified by mAbs 1A5 and 1B6 (Novocastra, UK), respectively. The goat polyclonal antibody N-19 to NKG2A was from Santa Cruz (CA, USA). Sections (5-μm thick) from routinely formalin-fixed (18-24 h) paraffin-embedded tissue blocks were stained by Immunohistochemistry (IHC). Antigen retrieval for HCA2, HC10, L31, CD8 and CD56 staining was carried out as described [23]. For MEM-E/02 and NKG2A staining, slides were dewaxed, rehydrated and heated in a water bath at 96°C for 40 minutes in EDTA (pH 8.0), followed by cooling at room temperature. Staining was revealed by a supersensitive streptavidin-biotin immunoperoxidase system (Biogenex, Menarini, Italy) for all antibodies. In addition, NKG2A staining was also revealed by a Goat AP-Polymer Kit (Biocare Medical, CA, USA). The chromogenic substrate was 3,3'-diaminobenzidine for all stainings. In addition, NKG2A staining was also revealed by Warp Red (Biocare Medical). Preliminary specificity assessments were performed on a panel of HLA-E-positive and HLA-E-negative cell lines [17] fixed and embedded as above. Different staining sessions included repeated staining of an internal normalization control (a section from a known specimen) to ensure day-to-day consistency. Because weak staining was invariably associated with the presence of negative areas, whereas strong staining tended to be homogeneous (this applies to both normal and tumor tissues), staining heterogeneity and staining intensity were factored into a single scale (termed *'absolute' *intensity score hereafter). The absolute intensity score may acquire one of four possible values: 0 (undetectable or faint in <20% of the cells); 1 (faint to weak in 20% but ≤70% of the cells); 2 (weak to moderate in >70% of the cells); 3 (intense in>70% of the cells). In analogy with our previously cited study [16], we calculated an additional intensity scale (termed *'relative' *hereafter) by subtracting the absolute score of the normal mucosa from that of its paired colon carcinoma specimen. Subtraction of two series of scores, each graded on a four-digit scale, resulted in the assignment of one of 7 possible algebraic values (*-3*, *-2*, *-1*, *0*, *+1*, *+2*, *+3*) to each normal-neoplastic pair. Intermediate (*-1*, *0*, and *+1*) and extreme (*-3*, *-2*, *+2*, and *+3*) relative scores identify pairs in which the tumor is similar to and deviates from, respectively, its normal paired mucosa. CD3, CD56 and NKG2A infiltrates were assessed in six randomly chosen high power fields (HPF, ×400), and expressed as either the percentage of positive cells in the total infiltrate, or the average number of positive cells in each HPF.

### Statistical analysis

Descriptive statistics were used to summarize pertinent study information. The Spearman rho correlation was used to determine relationships between parameters. The Hazard risk and the confidence limits were estimated for each variable using the Cox univariate model. Significance was defined at the *p *< 0.05 level. A multivariate Cox proportional hazard model was also developed using stepwise regression (forward selection) by selecting those predictive variables that were significant upon univariate analysis. Enter limit and remove limit were *p *= 0.10 and *p *= 0.15 respectively. Survival was calculated by the Kaplan-Meier product-limit method from the date of surgery until the time of death (overall survival; OS), relapse (disease-free survival; DFS), or last visit (OS and DFS), whichever applicable. The log-rank test was used to assess differences between subgroups. Significance was defined at the *p *< 0.05 level. Statistical analysis was carried out by the SPSS v. 13.0 (SPSS, Milan, Italy) software. Optimal cut-offs of NKG2A immunoreactivity defining two groups of patients with significantly different MEM-E/02 intensity scores were found using the Receiver Operating Characteristic (ROC) curve analysis.

### HLA-A, -B, -C typing

Genomic DNAs were extracted from the available frozen tumor tissue samples (*n *= 29), and typed for HLA-A, -B, and -C using sequence-specific primers (Olerup-SSP, Genovision, Vienna, Austria).

## Results

### Analysis of MEM-E/02 immunoreactivity in colorectal carcinomas and paired mucosa specimens

**Figure 1:**
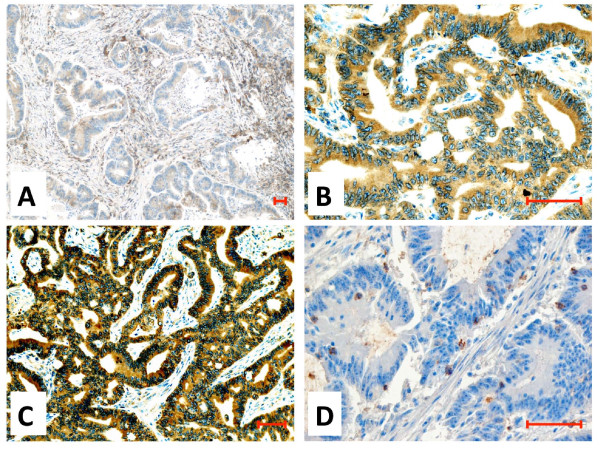
**Immunohistochemical staining of formalin-fixed, paraffin-embedded colorectal carcinoma tissues**. A-C: Representative reactivity scores of MEM-E/02 to HLA-E (see Materials and Methods): A = *1*, B = *2*, C = *3*. D: staining of lymphoid infiltrates with an antibody to the HLA-E-specific receptor NKG2A. Magnification: 10×, 40×, 20×, and 40× respectively. Scale bar = 100 μm.

Colorectal carcinomas from stage II/III patients (*n *= 149), each paired with morphologically normal colonic mucosa from the same patient, were stained by immunohistochemistry with the HLA-E-specific MEM-E/02 antibody. Representative examples of staining grades (recorded as *absolute scores*, see Materials and Methods) are provided in Figure 1A-C . HLA-E was detected in nearly all the specimens, approximately half of which displayed the highest absolute score of *3*. High absolute scores were most frequent also with HCA2 (to HLA-A), HC10 (to HLA-B) and L31 (to HLA-C), as shown in Additional file 1, Fig. S1A. Similar results were obtained by plotting the staining intensities of the normal mucosae (not shown). Thus, HLA-E is expressed in essentially all normal and neoplastic colonic tissues, often at high levels.

Next, we calculated the seven-digit *relative intensity scores *(see Materials and Methods) of paired immunohistochemical determinations. Representative results (Additional file 1, Fig. S1B) demonstrate that tumors resembling their normal paired mucosa (relative scores: *+1*, *0*, and *-1*) were far more numerous than tumors undergoing consistent up-regulation or down-regulation (relative scores: *-2*, *-3 *and *+2*, *+3*), a finding that applies to all the tested HLA class I molecules: HLA-E, HLA-A, HLA-B and HLA-C.

Finally, flow cytometry of 3 representative colorectal carcinoma cell lines provided evidence that at least some of the denatured HLA-A and HLA-E molecules detected by HCA2 and MEM-E/02 on archival tissues derive from native molecules that are co-expressed on the surface of live cells (Additional file 1, Fig. S2).

### Co-expression of HLA-E and permissive class I alleles

It has been shown that signal sequence-derived peptides from permissive alleles (most HLA-A and some HLA-C) carry methionine at P2, whereas peptides from non-permissive alleles (most HLA-B) carry threonine [24,25]. On this basis, absolute HLA-E levels are expected to correlate with (a) the levels of co-expressed products from at least some HLA-A, -B, and -C loci, and (b) the number of permissive alleles carried at the DNA level. To verify prediction (a), the staining intensities of MEM-E/02 (to HLA-E) were correlated with the staining intensities of HCA2, HC10, and L31 (preferentially reactive with HLA-A, HLA-B and HLA-C, respectively) in the 149 tumor specimens. A significant (Table 1, *p *= 0.01) correlation was detected between HLA-E and HLA-A, but not HLA-B or HLA-C. To verify prediction (b), 29 high-quality genomic DNAs (required for reliable allele-specific PCR) from colon carcinoma lesions were typed for HLA-A, -B, -C. From 3 to 6 permissive alleles (alleles and signal sequences listed at <http://hla.alleles.org/data/index.html>) were identified in each DNA. As in the complete case collection, HLA-E and HLA-A staining intensities did correlate also in this smaller subset (Table 1, *p *= 0.02; also see Additional file 1, Fig. S3A). In addition, there was a weak non-significant trend for correlation between the number of the putative HLA-A/-B/-C donor alleles and HLA-E staining intensity (Additional file 1, Fig. S3B). We conclude that HLA-E and at least its preferred ligand donors (from the HLA-A locus) are coordinately expressed in colorectal carcinoma lesions *in vivo*.

**Table 1:** Correlations among antibody reactivity scores

		**HCA2** **(HLA-A)**		**HC10** **(HLA-B)**		**L31** **(HLA-C)**	
		
		***r***	***p***	***r***	***p***	***r***	***p***
		
MEM-E/02	All specimens (*n *= 149)	**0.20**	**0.01**	0.13	0.11	0.07	0.28
		
	HLA-typed specimens (*n *= 29)	**0.42**	**0.02**	0.08	0.69	0.13	0.51

Significant correlations boldface.

### Association between HLA-E expression and survival

Next, univariate analysis and multivariate analysis of DFS and OS (Cox model) were performed on the entire series of 149 stage II/III colorectal cancer patients. In agreement with our previous study [16], advanced tumor stage and extreme up-/down-regulation of HCA2-reactive (HLA-A) molecules (relative scores of *+3*, *+2*, *-3*, and *-2*) were significant and independent predictors of both DFS and OS (Table 2, top two lines in both sections), whereas the absolute reactivity scores of HCA2, HC10 and L31 (Table 2, last three lines in both sections) as well as the relative reactivity scores of HC10 and L31 (not shown) were not. Contrary to HCA2, and quite surprisingly, the relative MEM-E/02 scale did not identify any significant prognostic category (not shown).

**Table 2:** Univariate and multivariate analyses of prognostic factors for disease-free and overall survival of stage II and III colon carcinoma patients

	DISEASE-FREE SURVIVAL			
	**Univariate Analysis**		**Multivariate Analysis**	
	
**Prognostic factors**	**HR^1 ^(95% CI) ^2^**	***p *value**	**HR (95% CI)**	***p *value**
Stage III vs II	2.55 (1.41-4.60)	**0.002**	2.77 (1.45-5.28)	**0.002**
HCA2 relative score (*+3*/*+2*/*-3*/*-2 *vs others)	2.50 (0.18-5.31)	**0.02**	2.94 (1.36-6.34)	**0.006**
MEM-E/02 absolute score (*0*/*1*/*2 *vs *3*)	1.90 (1.05-3.45)	**0.03**	1.82 (0.96-3.45)	**0.06**
T (3/4 vs 1/2)	1.26 (0.56-2.82)	0.58		
Grading (2/3 vs 1)	1.95 (0.47-8.04)	0.36		
HCA2 absolute score (*0*/*1 *vs *2*/*3*)	1.47 (0.77-2.79)	0.25		
HC10 absolute score (*2*/*3 *vs *0*/*1*)	2.40 (0.74-7.74)	0.14		
L31 absolute score (*0*/*1 *vs *2*/*3*)	1.36 (0.73-2. 57)	0.33		

	**OVERALL SURVIVAL**			

	**Univariate Analysis**		**Multivariate Analysis**	
	
**Prognostic factors**	**HR^1 ^(95% CI) ^2^**	***p *value**	**HR (95% CI)**	***p *value**
Stage III vs II	2.78 (1.45-5.34)	**0.002**	2.49 (1.23-5.04)	**0.01**
HCA2 relative score (*+3*/*+2*/*-3*/*-2 *vs others)	3.09 (1.43-6.69)	**0.004**	3.55 (1.61-7.87)	**0.002**
MEM-E/02 absolute score (*0*/*1*/*2 *vs *3*)	1.96 (1.01-3.81)	**0.05**	1.81 (0.90-3.63)	**0.09**
T (3/4 vs 1/2)	1.41 (0.55-3.61)	0.48		
Grading (2/3 vs 1)	1.57 (0.38-6.52)	0.54		
HCA2 absolute score (0/1 vs 2/3)	1.66 (0.83-3.31)	0.15		
HC10 absolute score (*2*/*3 *vs *0*/*1*)	1.91 (0.59-6.22)	0.28		
L31 absolute score (*0*/*1 *vs *2*/*3*)	1.21 (0.60-2.44)	0.61		

**^1 ^**HR: hazard risk

**^2 ^**CI: 95% confidence interval

Significant correlations boldface

**Figure 2:**
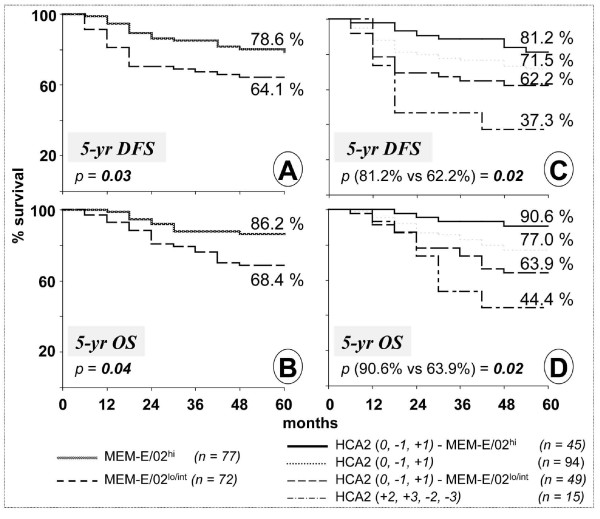
**Kaplan-Meier survival curves of patients with colorectal carcinoma**. Five-year disease-free (A) and overall (B) survival rates of 149 stage II-III colorectal cancer patients according to the MEM-E/02 (HLA-E) absolute immunohistochemical score (MEM-E/02^hi^= *3*; MEM-E/02^lo/int^: *0*, *1 *and *2*). Five-year disease-free (C) and overall (D) survival of stage II-III colon carcinoma patients according to the HCA2 (HLA-A) relative immunohistochemical score (*0*, *-1*, *+1 *vs *+2*, *+3*, *-2*, *-3*). Patients (*n *= 94) with an HCA2 reactivity mimicking that seen in the normal paired mucosa (relative scores *0*, *-1 *and *+1*) were sorted into MEM-E/02^hi ^(absolute reactivity score = *3*) and MEM-E/02^lo/int ^(*0*, *1*, and *2*). The percent survival, shown in ordinate, is analytically displayed for the different groups at the end of the observation period, along with the statistical significance (*p*) of the observations.

However, a high MEM-E/02 absolute staining intensity score of *3 *did display a significant correlation with favorable prognosis upon both univariate analysis and multivariate analysis (Table 2, third line from top in both sections). Accordingly, Kaplan-Meier analysis (Figure 2A and 2B) was consistent with significantly longer DFS and OS in patients bearing tumors with high levels of absolute MEM-E/02 reactivity (HLA-E^hi^).

Next, we investigated whether a combined evaluation of relative HCA2 reactivity and absolute MEM-E/02 reactivity might improve the accuracy of outcome prediction. In the patients with unfavorable HCA2 phenotypes (extreme HLA-A up-/down-regulation), absolute HLA-E expression did not significantly associate with 5-year DFS and OS (not shown), suggesting that 'abnormal' HCA2 reactivity is a dominant prognostic factor, and the influence of HLA-E in this tumor subset is negligible. In contrast (Figure 2C and 2D), among patients bearing tumors with HLA-A levels similar to that of the normal autologous mucosa (that are by themselves associated with favorable prognosis), high and low/intermediate HLA-E expression levels further discriminated two numerically equivalent populations (*n *= 49 vs 45) significantly differing in their chances of relapse and death within 5 years.

### Correlation between HLA-E expression in tumors and NKG2A expression in lymphoid infiltrates

**Figure 3:**
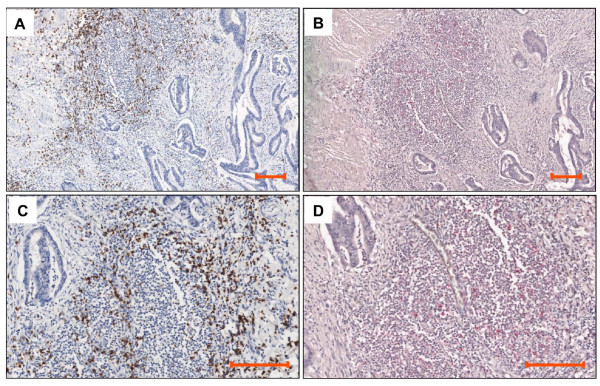
**Immunohistochemical staining for CD8 and NKG2A**. Two consecutive sections of a colorectal carcinoma lesion displaying a strongly and homogeneously positive (MEM-E/02 score = *3*) neoplastic mucosa were stained by mAb 1A5 to CD8 (A and C) and the polyclonal antibody N-19 to NKG2A (B and D). Different chromogens reveal CD8 and NKG2A cells in a large peritumoral infiltrate. Counterstaining with Haematoxilin. Magnification 10 × (panel A and B) and 20 × (panel C and D). Scale bar = 100 μm.

Sixteen lesions expressing the highest HLA-E levels (MEM-E/02 score = *3*), and 12 lesions expressing the lowest levels (score = *1*) were assessed for NKG2A (Figure 1D), CD8 and CD56 infiltrates (not shown). Up to 5% and 20% of colorectal carcinoma infiltrates were reactive with NKG2A and CD8, respectively. In contrast, only scattered CD56 cells (no more than 2 per microscopic field) were detected. As shown by a representative stain of serial sections (Figure 3), the distribution of NKG2A-positive and CD8-positive cells partly overlaps.

Upon ROC analysis there was no significant correlation between CD8 infiltrate (either the extent or number of positive cells) and HLA-E expression. In contrast, high and low levels of the HLA-E ligand in tumor tissues correlated with high and low numbers, respectively, of infiltrating lymphoid cells expressing its specific receptor NKG2A (Additional file 1, Table S1; *p *= 0.04).

## Discussion

Although intracellular transport of HLA-E is believed to be quite inefficient [26], colorectal carcinoma cells (like most neoplastic cell lines) co-express on the surface HLA-E and classical HLA class I molecules, including HLA-A ([18] and Additional file 1, Fig. S2).

The present immunohistochemical study of colorectal carcinoma lesions with the MEM-E/02 antibody [17] reveals that high expression of HLA-E significantly correlates with high expression of HLA-A, but not HLA-B or HLA-C. Since HLA-E ligands donated by most HLA-B and at least some HLA-C alleles are poorer NKG2A inhibitors than those donated by HLA-A alleles [27], *in vitro *mechanisms of ligand donation and HLA-E stabilization appear to be recapitulated *in vivo*.

Ligand donation by permissive HLA class I alleles is a well-known strategy to signal integrity of the so-called antigen processing machinery, and prevent inappropriate immune lysis upon HLA-E:NKG2A inhibition [1,2]. Consistent with HLA-E enabling active scrutiny by the immune system (rather than immunoevasive responses), we show herein that HLA-E^hi ^phenotypes are associated with a lymphoid infiltrate (mainly CD8) enriched in the HLA-E-specific inhibitory receptor NKG2A, and a favorable prognosis.

Similar to previous studies [28,29], in which the extent of the colorectal carcinoma lymphoid infiltrate was correlated with a favorable prognosis, only scattered NK cells were detected herein. However, this does not obviously rule out a role of infiltrating NK cells *in vivo*.

Interestingly, we found that high HLA-E expression is associated *per se *with a favorable outcome, but it is the combination between high HLA-E (on an absolute scale) and similar HLA-A levels in the tumor compared to the normal mucosa (on a relative expression scale) that results in the precise identification of graded levels of prognosis.

Also of note, HLA-A clearly dominates the prognostic landscape both in quantitative and qualitative terms. Quantitatively, HLA-A identifies a wider prognostic gap between favorable and poor outcomes. Qualitatively, HLA-A was shown previously to be an early-stage prognosticator [16], whereas HLA-E is shown herein to influence prognosis particularly within a cohort of patients pre-selected for a favorable outcome through HLA-A phenotyping.

**Figure 4:**
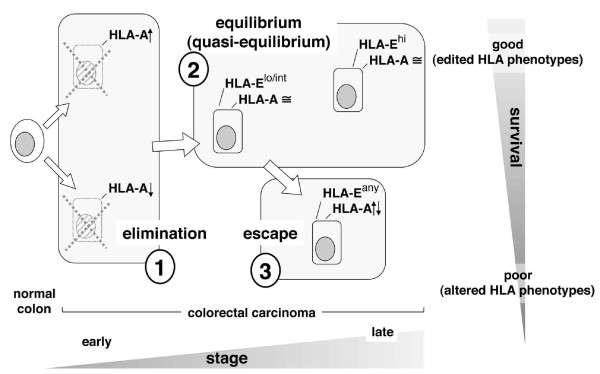
**Class I HLA expression, immunoediting, and survival through colorectal carcinoma stages**. Stepwise selection of HLA phenotypes results in distinct tumor variants populating the three classical steps (elimination, equilibrium and escape; shaded boxes) of immunoediting. Equilibrium is defined herein quasi-equilibrium. Unlike 'true' equilibrium, that corresponds to occult cancer and dormancy [30], and was elusive even in experimental models of mouse tumorigenesis [39], quasi-equilibrium refers to edited tumor cells embedded in a clinically evident tumor mass, sufficiently large to be immunophenotyped in a human pathology facility.

The easiest interpretation of these results involves the assignment of the observed HLA-A and HLA-E phenotypic variants to the three classical phases (elimination, equilibrium and escape; boxes n. 1, 2 and 3 in Figure 4) of the widely accepted 'immunoediting' theory. This theory was originally proposed by Dunn et al.[30] to interpret murine models of tumor escape. We are not aware of previous attempts to classify specific class I HLA phenotypes in the context of stepwise immunoediting. We propose that in the first immunoediting phase (box 1, elimination), HLA-A^hi ^and HLA-A^lo ^phenotypes are suppressed, for instance by anti-tumor CTL and NK cells equipped with functionally dominant activating and inhibitory receptors, respectively. As described by us several years ago [13,14], this results in the selection of 'low profile' (HLA-A phenotypes, e.g. colorectal carcinoma cells expressing HLA-A levels similar to those of their normal counterparts. Next, two phenotypes arise characterized by different HLA-E levels (box 2, immunoediting): HLA-A/HLA-E^lo/int ^and HLA-A/HLA-E^hi^. Low/intermediate and high HLA-E levels are hypothesized to result: (a) the former from limited ligand donation and HLA-E stabilization, as a result of HLA-A^hi ^suppression during the previous elimination phase; (b) the latter from HLA-A-independent events of ligand donation. Why both might incite anti-tumor immune responses, at two different levels, could be explained by relief of lytic inhibition and/or triggering immune responses. There are precedents for both. Relief of inhibition by competitive binding of non-HLA-derived ligands is known to favor NK lysis [4]. Direct triggering of cytotoxic CD8 lymphocytes has been described to result from the presentation of a non-conventional tumor antigen in the context of an undefined non-classical class I molecule [31].

Finally (box 3, escape), extreme HLA-A phenotypes (HLA-A↑↓)1, very different from those seen in the normal mucosa, and initially suppressed during the elimination phase, may (re)appear. Having forced all the immune checkpoints, these phenotypes are not surprisingly associated with the poorest outcomes regardless of HLA-E expression, found to be irrelevant in this group.

Thus, we propose a dual outcome model whereby HLA-E expression, far from being a manifestation of immune inhibition and escape, represents instead a prerequisite for active immunological scrutiny of edited HLA tumor variants on the one hand, and triggering immune responses on the other.

Recently, Levy et al. reported poor MEM-E/02 reactivity in most colorectal carcinomas, and an association between high HLA-E expression, detected in just 8 lesions, and poor prognosis [32]. Poor reactivity is difficult to reconcile with the high levels of HLA-E detected by us with the same MEM-E/02 antibody in most lesions, and the prognostic associations proposed by Levy et al. are based on a limited number of cases. Another example of HLA-E association with poor prognosis is a recent study on early-stage breast carcinoma [33].

Our results better agree with two cDNA profiling studies of advanced melanoma in which high levels of HLA-E transcripts contributed molecular signatures of favourable prognosis [34,35], and a recent study on glioblastoma [36]. Possibly, the balance between activating and inhibitory functions of HLA-E may differ in different tumor histotypes. Additional work is required to investigate these issues in the context of activation-inhibition models of tumor immune surveillance.

## Conclusions

In summary, this is one of the few reports [32,33,37] addressing the prognostic significance of non-classical class I molecules in colorectal carcinoma. Additionally, we provide evidence (albeit indirect) for a functional interplay between classical and non-classical HLA molecules *in vivo*. This is also the first report to suggest *in situ *engagement of tumor cells and T lymphocytes expressing a defined ligand:non-rearranging immune receptor pair.

## Note added in proof

At the time of proof-editing a paper [38] appeared in PubMed describing a poor prognosis associated with HLA-E expression in colorectal carcinoma and NKG2A expression in the lymphoid infiltrate.

## Competing interests

The authors declare that they have no competing interests.

## Authors' contributions

MC provided surgical specimens and supervised patient follow-up. MM and MC organized the tissue bank and the clinical pathological database. MGD reviewed and staged pathological specimens. MB, MM, CE and FR performed and evaluated immunohistochemistry. ET and ELM prepared the antibodies and performed flow cytometry and molecular HLA typing. IS participated in the design of the study and performed statistical analysis. MB, MM, MC and PG conceived, designed, and coordinated the study. MB and PG wrote the manuscript. All authors read and approved the final manuscript.

## Supplementary Material

Additional file 1 **Supplemental materials**. Additional Tables, Figures, and Legends [7,17,20,40,41]. Click here for file

## References

[B1] Braud VM Allan DSJ O'Callaghan CA Söderström K D'Andrea A Ogg GS Lazetic S Young NT Bell JI Phillips JH Lanier LL McMichael AJ HLA-E binds to natural killer cell receptors CD94/NKG2A, B and C Nature 1998 391 795 799 10.1038/35869 9486650

[B2] Rodgers J Cook R MHC class Ib molecules bridge innate and acquired immunity Nat Rev Immunol 2005 5 459 471 10.1038/nri1635 15928678

[B3] Moretta L Romagnani C Pietra G Moretta A Mingari MC NK-CTLs, a novel HLA-E-restricted T-cell subset Trends Immunol 2003 24 136 143 10.1016/S1471-4906(03)00031-0 12615209

[B4] Michaelsson J Teixeira de Matos C Achour A Lanier LL Kärre K Soderstrom K A signal peptide derived from hsp60 binds HLA-E and interferes with CD94/NKG2A recognition J Exp Med 2005 196 1403 1414 10.1084/jem.20020797 PMC2194258 12461076

[B5] Cohen GB Gandhi RT Davis DM Mandelboim O Chen BK Strominger JL Baltimore D The selective downregulation of class I major histocompatibility complex proteins by HIV-1 protects HIV-infected cells from NK cells Immunity 1999 10 661 671 10.1016/S1074-7613(00)80065-5 10403641

[B6] Smith MEF Bodmer WF Bodmer JG Selective loss of HLA-A, B, C locus products in colorectal adenocarcinoma Lancet 1988 I 823 824 10.1016/s0140-6736(88)91682-0 2895339

[B7] Lee N Goodlett DR Ishitani A Marquardt H Geraghty DE HLA-E surface expression depends on binding of TAP-dependent peptides derived from certain HLA class I signal sequences J Immunol 1998 160 4951 4960 9590243

[B8] Tomasec P Braud VM Rickards C Powell MB McSharry BP Gadola S Cerundolo V Borysiewicz LK McMichael AJ Wilkinson GWG Surface expression of HLA-E, an inhibitor of natural killer cells, enhanced by human cytomegalovirus gpUL40 Science 2000 287 1031 1033 10.1126/science.287.5455.1031 10669413

[B9] Ulbrecht M Martinozzi S Grzeschik M Hengel H Ellwart JW Pla M Weiss EH The human cytomegalovirus UL40 gene product contains a ligand for HLA-E and prevents NK cell-mediated lysis J Immunol 2000 164 5019 5022 10799855 10.4049/jimmunol.164.10.5019

[B10] Gudmundsdóttir I Jónasson JG Sigurdsson H Olafsdóttir K Tryggvadóttir L Ogmundsdóttir HM Altered expression of HLA class I antigens in breast cancer: Association with prognosis Int J Cancer 2000 89 500 505 10.1002/1097-0215(20001120)89:6<500::AID-IJC6>3.0.CO;2-# 11102894

[B11] Menon AG Morreau H Tollenaar RA Alphenaar E Van Puijenbroek M Putter H Janssen-Van Rhijn CM Van De Velde CJ Fleuren GJ Kuppen PJ Down-regulation of HLA-A expression correlates with a better prognosis in colorectal cancer patients Lab Invest 2002 82 1725 1733 12480922 10.1097/01.lab.0000043124.75633.ed

[B12] Watson NF Ramage JM Madjd Z Spendlove I Ellis IO Scholefield JH Durrant LG Immunosurveillance is active in colorectal cancer as downregulation but not complete loss of MHC class I expression correlates with a poor prognosis Int J Cancer 2005 118 6 10 10.1002/ijc.21303 16003753

[B13] Giacomini P Giorda E Fraioli R Nicotra MR Vitale N Setini A Delfino L Morabito A Benevolo M Venturo I Mottolese M Ferrara GB Natali PG Low prevalence of selective human leukocyte antigen (HLA)-A and HLA-B epitope losses in early-passage tumor cell lines Cancer Res 1999 59 2657 2667 10363989

[B14] Giorda E Sibilio L Martayan A Moretti S Venturo I Mottolese M Ferrara GB Cappellacci S Eibenschutz L Catricalà C Grammatico P Giacomini P The antigen processing machinery of Human Leukocyte Antigens: linked patterns of gene expression in neoplastic cells Cancer Res 2003 63 4119 4127 12874016

[B15] Fruci D Ferracuti S Limongi MZ Cunsolo V Giorda E Fraioli R Sibilio L Carroll O Hattori A van Endert PM Giacomini P Expression of endoplasmic reticulum aminopeptidases in EBV-B cell lines from healthy donors and in leukemia/lymphoma, carcinoma, and melanoma cell lines J Immunol 2006 176 4869 4879 16585582 10.4049/jimmunol.176.8.4869

[B16] Benevolo M Mottolese M Piperno G Sperduti I Cione A Sibilio L Martayan A Donnorso RP Cosimelli M Giacomini P HLA-A, -B, -C expression in colon carcinoma mimics that of the normal colonic mucosa and is prognostically relevant Am J Surg Pathol 2007 31 76 84 10.1097/01.pas.0000213343.55605.b9 17197922

[B17] Lo Monaco E Sibilio L Melucci E Tremante E Suchànek M Horejsi V Martayan A Giacomini P HLA-E: strong association with β2m and surface expression in the absence of HLA class I signal sequence-derived peptides J Immunol 2008 181 5442 5450 18832701 10.4049/jimmunol.181.8.5442

[B18] Lo Monaco E Tremante E Cerboni C Melucci E Sibilio L Zingoni A Nicotra MR Natali PG Giacomini P Human Leukocyte Antigen E contributes to protect tumor cells from lysis by natural killer cells Neoplasia 2011 13 822 830 21969815 10.1593/neo.101684 PMC3182274

[B19] TNM classification of malignant tumors 2002 6 Hoboken, NJ: John Wiley & Sons

[B20] Stam NJ Vroom TM Peters PJ Pastoors EB Ploegh HL HLA-A and HLA-B-specific monoclonal antibodies reactive with free heavy chains in Western blots, in formalin-fixed, paraffin-embedded tissue sections and in cryoimmuno-electron microscopy Int Immunol 1990 2 113 125 10.1093/intimm/2.2.113 2088481

[B21] Seitz C Uchanska-Ziegler B Zank A Ziegler A The monoclonal antibody HCA2 recognises a broadly shared epitope on selected classical as well as several non-classical HLA class I molecules Mol Immunol 1998 35 819 827 10.1016/S0161-5890(98)00077-7 9839550

[B22] Setini A Beretta A De Santis C Meneveri R Martayan A Mazzilli MC Appella E Siccardi AG Natali PG Giacomini P Distinctive features of the α1 domain α helix of HLA-C heavy chains free of β2-microglobulin Hum Immunol 1996 46 69 81 10.1016/0198-8859(96)00011-0 8727205

[B23] Giacomini P Beretta A Nicotra MR Ciccarelli G Martayan A Cerboni C Lopalco L Bini D Delfino L Ferrara GB Siccardi AG Natali PG HLA-C heavy chains free of β2m: distribution in normal tissues and neoplastic lesions of non-lymphoid origin and IFN-γ responsiveness Tissue Antigens 1997 50 555 566 10.1111/j.1399-0039.1997.tb02913.x 9458108

[B24] Braud VM Jones EY McMichael A The human major histocompatibility complex class Ib molecule HLA-E binds signal sequence-derived peptides with primary anchor residues at positions 2 and 9 Eur J Immunol 1997 27 1164 1169 10.1002/eji.1830270517 9174606

[B25] Borrego F Ulbrecht M Weiss EH Coligan JE Brooks AG Recognition of human histocompatibility leukocyte antigen (HLA)-E complexed with HLA class I signal sequence-derived peptides by CD94/NKG2 confers protection from natural killer cell-mediated lysis J Exp Med 1998 187 813 818 10.1084/jem.187.5.813 9480992 PMC2212178

[B26] Ulbrecht M Kellermann J Johnson JP Weiss EH Impaired intracellular transport and cell surface expression of nonpolymorphic HLA-E: evidence for inefficient peptide binding J Exp Med 1992 176 1083 1090 10.1084/jem.176.4.1083 1402654 PMC2119380

[B27] Brooks AG Borrego F Posch PE Patamawenu A Scorzelli CJ Ulbrecht M Weiss EH Coligan JE Specific recognition of HLA-E, but not classical, HLA class I molecules by soluble CD94/NKG2A and NK cells J Immunol 1999 162 305 313 9886400

[B28] Galon J Costes A Sanchez-Cabo F Kirilovsky A Mlecnik B Lagorce-Pages C Tosolini M Camus M Berger A Wind P Zinzindohoue F Bruneval P Cugnenc PH Trajanoski Z Fridman WH Pages F Type, density, and location of immune cells within human colorectal tumors predict clinical outcome Science 2006 313 1960 1964 10.1126/science.1129139 17008531

[B29] Ohtani H Focus on TILs: prognostic significance of tumor infiltrating lymphocytes in human colorectal cancer Cancer Immun 2007 7 4 12 17311363 PMC2935759

[B30] Dunn GP Bruce AT Ikeda H Old LJ Schreiber RD Cancer immunoediting: from immunosurveillance to tumor escape Nat Immunol 2002 3 991 998 10.1038/ni1102-991 12407406

[B31] Housseau F Bright RK Simonis T Nishimura MI Topalian SL Recognition of a shared human prostate cancer-associated antigen by nonclassical MHC-restricted CD8+ T cells J Immunol 1999 163 6330 6337 10570328

[B32] Levy EM Bianchini M Von Euw EM Barrio MM Bravo AI Furman D Domenichini E Macagno C Pinsky V Zucchini C Valvassori L Mordoh J Human leukocyte antigen-E protein is overexpressed in primary human colorectal cancer Int J Oncol 2008 32 633 641 18292941

[B33] de Kruijf EM Sajet A van Nes JG Natanov R Putter H Smit VT Liefers GJ van den Elsen PJ van de Velde CJ Kuppen PJ HLA-E and HLA-G Expression in Classical HLA Class I-Negative Tumors Is of Prognostic Value for Clinical Outcome of Early Breast Cancer Patients J Immunol 2010 185 7452 7459 10.4049/jimmunol.1002629 21057081

[B34] John T Black MA Toro TT Leader D Gedye CA Davis ID Guilford PJ Cebon JS Predicting clinical outcome through molecular profiling in stage III melanoma Clin Cancer Res 2008 14 5173 5180 10.1158/1078-0432.CCR-07-4170 18698035

[B35] Mandruzzato S Callegaro A Turcatel G Francescato S Montesco MC Chiarion-Sileni V Mocellin S Rossi CR Bicciato S Wang E Marincola FM Zanovello P A gene expression signature associated with survival in metastatic melanoma J Transl Med 2006 4 50 10.1186/1479-5876-4-50 17129373 PMC1697826

[B36] Kren L Slaby O Muckova K Lzicarova E Sova M Vybihal V Svoboda T Fadrus P Lakomy R Vanhara P Krenova Z Sterba J Smrcka M Michalek J Expression of immune-modulatory molecules HLA-G and HLA-E by tumor cells in glioblastomas: An unexpected prognostic significance? Neuropathology 2011 31 129 134 10.1111/j.1440-1789.2010.01149.x 20667016

[B37] Ye SR Yang H Li K Dong DD Lin XM Yie SM Human leukocyte antigen G expression: as a significant prognostic indicator for patients with colorectal cancer Mod Pathol 2007 20 375 383 10.1038/modpathol.3800751 17277760

[B38] Bossard C Bezieau S Matysiak-Budnik T Volteau C Laboisse CL Jotereau F Mosnier JF HLA-E/beta2 microglobulin over-expression in colorectal cancer is associated with recruitment of inhibitory immune cells and tumor progression Int J Cancer 2011 10.1002/ijc.26453 21953582

[B39] Koebel CM Vermi W Swann JB Zerafa N Rodig SJ Old LJ Smyth MJ Schreiber RD Adaptive immunity maintains occult cancer in an equilibrium state Nature 2007 450 903 907 10.1038/nature06309 18026089

[B40] Cifaldi L Lo Monaco E Forloni M Giorda E Lorenzi S Petrini S Tremante E Pende D Locatelli F Giacomini P Fruci D NK cells efficiently reject lymphoma silenced for the endoplasmic reticulum aminopeptidase associated with antigen processing Cancer Res 2011 10.1158/0008-5472.CAN-10-3326 21252114

[B41] Bicknell DC Rowan A Bodmer WF β2-microglobulin mutations: a study of established colorectal cell lines and fresh tumors ProcNatlAcadSci 1994 91 4751 4755 10.1073/pnas.91.11.4751 PMC43866 8197130

