# Supplementary material for: High expression of HLA-E in colorectal carcinoma is associated with a favorable prognosis
Source: J Transl Med. 2011 Oct 27;9:184. doi: 10.1186/1479-5876-9-184 (PMC3219584; doi:10.1186/1479-5876-9-184)
Supplement: Additional file 1 — Supplemental materials. Additional Tables, Figures, and Legends [7,17,20,40,41]. [file 1479-5876-9-184-S1.PDF]

High expression of HLA-E in colorectal carcinoma is associated with a favorable prognosis

Maria Benevolo et al.

Supplemental Materials

TABLE S1 – Correlation between the MEM-E/02 (HLA-E) reactivity of colorectal carcinoma and the NKG2A reactivity of the corresponding lymphoid infiltrates

		MEM-E/02 absolute score	
		1	*3*
Mean n° of NKG2A positive cells *per* HPF	≤6	6[1] (75.00%)	2 (25.00%)
	>6	6 (30.00%)	14 (70.00%)

***p* = 0.04**[2]

[1] Number of cases
[2] Calculated by ROC analysis (see Materials and Methods)

Legends to Supplemental Figures

Figure S1

Distribution of staining intensity scores. The percentage of tumor specimens displaying any given absolute (A) and relative (B) intensity score is displayed for the indicated antibodies.

Figure S2

To provide the proof of principle that HCA2 and MEM-E/02 detect, in immunohistochemistry, HLA-A and HLA-E molecules that are co-expressed on the cell surface, representative colorectal carcinoma cell lines (HT-29 and Caco 2, from the American Type Culture Collection) were assessed by flow cytometry. Because HCA2 and MEM-E/02 detect linear epitopes mostly hidden in conformed, surface-expressed class I heavy chains [17, 20], surface antigens were denatured by short (3 min) incubation of colorectal carcinoma cells in 250 μl of 300 mM glycine HCl/ 1% Bovine Serum Albumin pH 2.5 on ice[40] . Following neutralization by dilution in 10 ml of RPMI 1640 culture medium containing 10% Fetal Bovine Serum, the cells were stained with HCA2 and MEM-E/02 as well as control antibodies to conformational heavy chain class I epitope (W6/32) and the associated light chain subunit β_2m (Namb-1), that are instead expected to be lost upon low-pH-induced unfolding and/or β_2m dissociation. 221.AEH lymphoblastoid cells (a generous gift of Dr. Daniel Geraghty) and colorectal carcinoma LoVo cells were also included as controls. The former express a chimeric HLA-E molecule capable of surface expression as a result of self ligand donation [7]. The latter lack β_2m and express little if any surface class I heavy chains [41]. As expected, acid treatment resulted in 50% to 75% reduction in W6/32 and Namb-1 binding, and in 33% to 50% increase in HCA2 and MEM-E/02 binding. Thus, HCA2 and MEM-E/02 reactivity of archival material (fixed in formalin and embedded in paraffin) is likely to reveal locus-specific linear epitopes hidden on a large set of native molecules, including those that are co-expressed on the cell surface.

Figure S3

Correlation in the expression of HLA-E and permissive HLA-A alleles. The absolute staining intensities of MEM-E/02 were plotted vs. (A) the absolute staining intensities of HCA2 (4-digit scales in both cases), and (B) the number of permissive alleles carried by a subset of 29 colorectal carcinomas typed for HLA-A, -B, -C. Each dot represents the scattering of two paired values from an individual lesion. Regression lines, *r* values, and *p* values are shown (n.s.: nonsignificant). Data in (A) are also summarized in Table I.

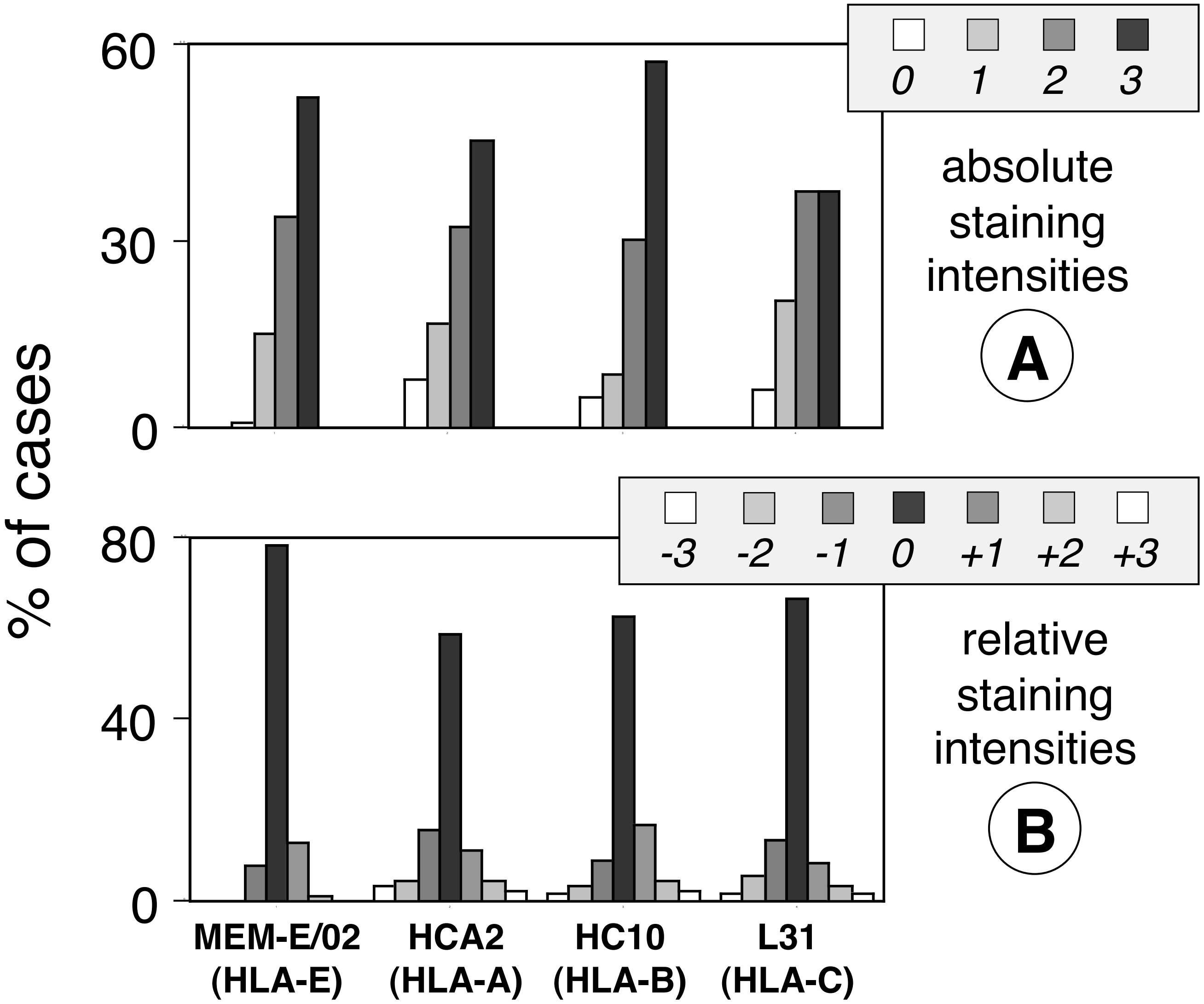

60
30
0
% of cases
0
1
2
3
absolute staining intensities
A
80
40
0
-3
-2
-1
0
+1
+2
+3
relative staining intensities
B
MEM-E/02 (HLA-E)
HCA2 (HLA-A)
HC10 (HLA-B)
L31 (HLA-C)

Fig. S1

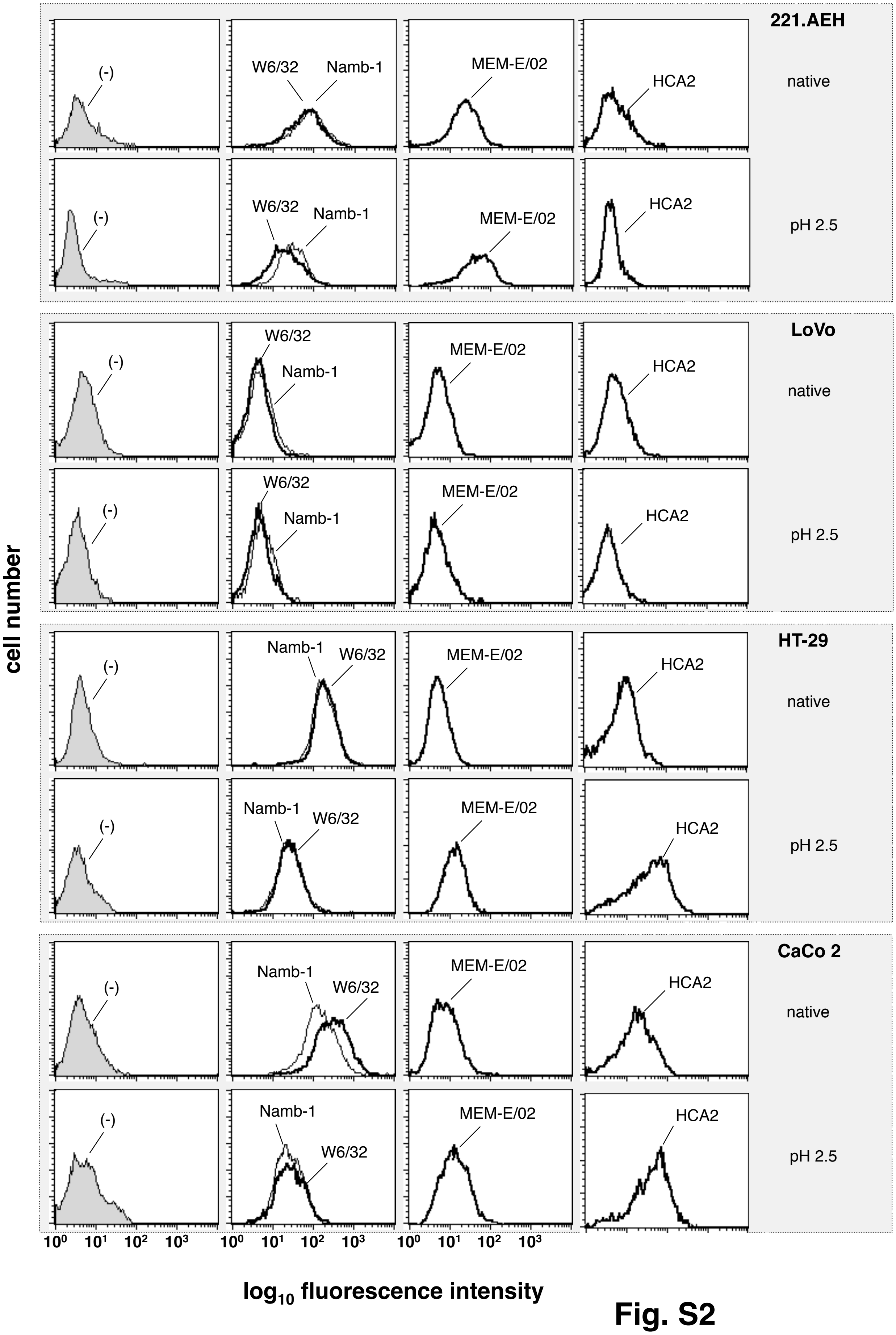
221.AEH
native
pH 2.5
LoVo
HT-29
CaCo 2
(-)
W6/32
Namb-1
MEM-E/02
HCA2
cell number
10⁰ 10¹ 10² 10³
log10 fluorescence intensity

Fig. S2

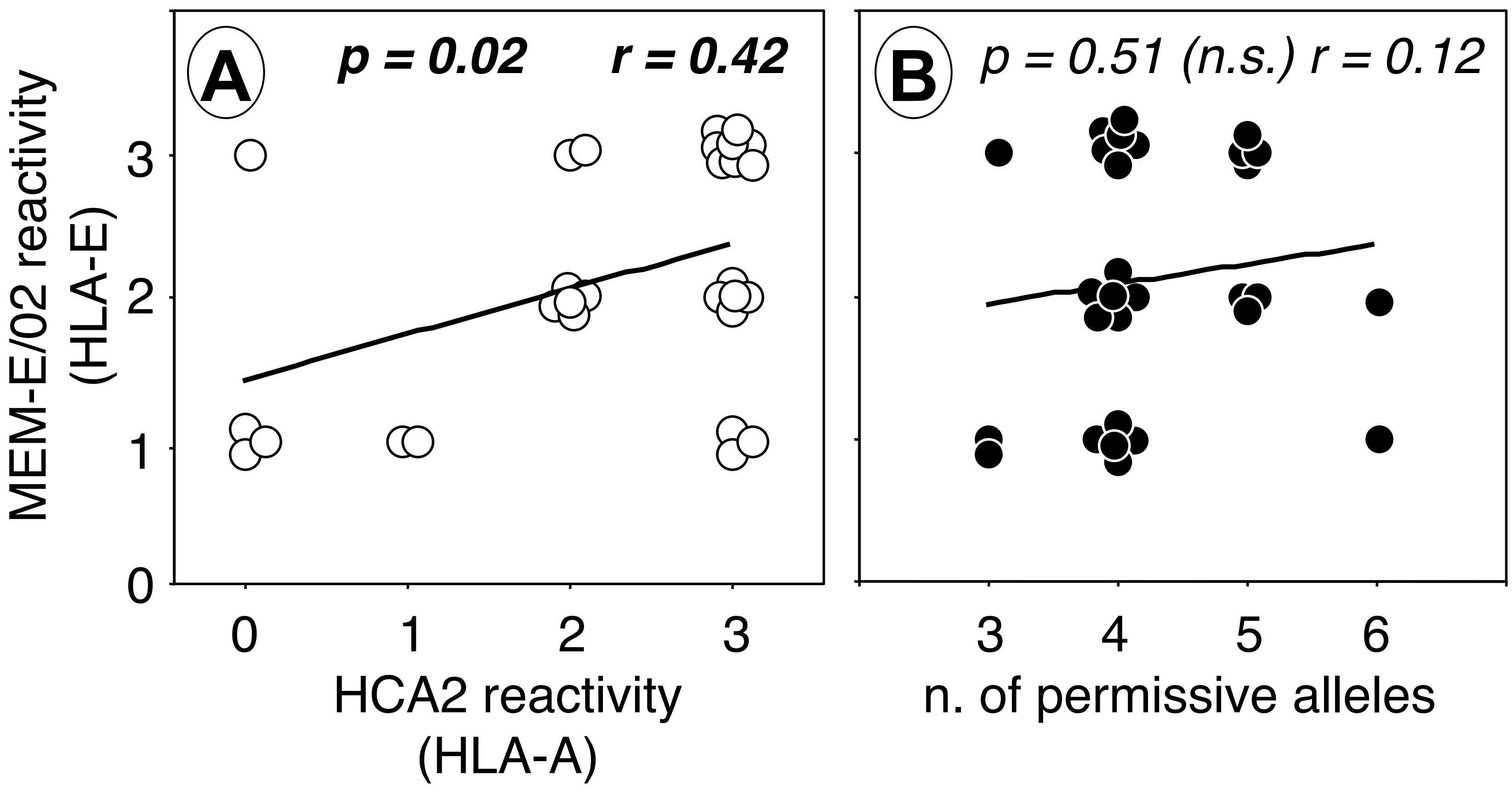
A
p = 0.02
r = 0.42
B
p = 0.51 (n.s.) r = 0.12
MEM-E/02 reactivity
(HLA-E)
0
1
2
3
0
1
2
3
HCA2 reactivity
(HLA-A)
3
4
5
6
n. of permissive alleles

Fig. S3
